# pH-Sensitive Membranes with Smart Cleaning Capability for Efficient Emulsion Separation and Pollutant Removal

**DOI:** 10.3390/membranes11030193

**Published:** 2021-03-11

**Authors:** Jiaming Zhang, Xiansheng Zhang, Wei Wei, Huiling Zhang, Yunfei Wang, Guoqiang Cai, Jindan Wu

**Affiliations:** 1 MOE Key Laboratory of Advanced Textile Materials & Manufacturing Technology, Zhejiang Sci-Tech University, Hangzhou 310018, China; zjm267798@163.com (J.Z.); zhangxianshengbaby@163.com (X.Z.); ryderwei@163.com (W.W.); zhl19991001@163.com (H.Z.); wyf1448618557@163.com (Y.W.); 2 NICE Zhejiang Technology Co. Ltd., Hangzhou 310013, China

**Keywords:** oil/water separation, surface charge, antifouling, dye removal, water purification

## Abstract

Since anionic dyes and surfactants abundantly exist in oily wastewater, both the separation of oil/water mixture and removal of low-molecular-weight pollutants are important to realize the advanced purification of water. By grafting poly(2-dimethylaminoethyl methacrylate) (pDMAEMA) onto polyethylene (PP) membrane via ultraviolet (UV)-initiated polymerization, the obtained PP-*g*-pDMAEMA membrane presented positively in water and negatively in an alkaline buffer (pH 9.0), respectively. Due to the switchable surface charge, the membrane had high emulsion separation efficiency and flux recovery ratio (approximately 100%). Besides, the dye (reactive black 5, RB-5) adsorption capacity reached 140 mg/m^2^ in water, and approximately 90% RB-5 could be released in pH 9.0. The anionic surfactant (sodium dodecyl benzene sulfonate, SDBS) was also reversely interpreted and released by the membrane via manipulating the ambient pH. The membrane constructed in this study is supposed to realize emulsion separation with smart cleaning capability, as well as the removal of dyes and surfactants, which could be utilized for multifunctional water purification.

## 1. Introduction

With the development of industrial society, the discharge of a large amount of oily wastewater has caused serious problems to the environment [1,2]. The oil/water separation technology has received great attention in the past decades. Among all the technologies, membrane separation is an efficient way for oily wastewater treatment and has already shown a good application prospect [3]. For example, a superhydrophilic and underwater superoleophobic poly(vinylidene fluoride) (PVDF) membrane prepared by salt-induced phase inversion can effectively separate both surfactant-free and surfactant-stabilized oil/water mixtures driven by gravity [4]. Although the membrane with specific physicochemical properties is proven to be effective in emulsion separation, poor antifouling performance still limits their widespread application [5]. Furthermore, the contaminated membranes abandoned may cause secondary pollution since they are difficult to degrade in nature [6].

Since sewage always contains oils and impurities such as organic dyes, surfactants, and microorganisms, the membrane is easily blocked by the pollutants and thereby loses its separation performance quickly in use. Zhao et al. prepared a hybrid polyethersulfone (PES) membrane by blending silver nanoparticles (AgNPs), halloysite nanotubes (HNTs), and graphene oxide (rGO) nanocomposite into the matrix [7]. The hybrid membrane exhibited superior hydrophilicity and surface smoothness than the pure PES one. However, the flux still reduced 30%~40% after one hour of operation. To simultaneously improve the separation efficiency and long-term stability has become a key challenge for membrane technology.

Generally, the membrane cannot eliminate the pollutants by itself, leading to gradual reduction of membrane flux. To solve this problem, a membrane with switchable surface wettability has been developed to achieve intelligent decontamination [8,9,10]. Smart polymers could change their properties by responding to external stimuli including temperature [11], light [12], pH [13], magnetic field [14], and so on. For example, the matrix coated with polyaniline (PANI) switched its super-wettability by responding to pH and released oil in high pH to achieve excellent recyclability [15]. In addition to the surface hydrophilicity, the surface charge also has a remarkable influence on the membrane function [16]. However, the relationship between surface charge and separation performance of membrane has not been investigated adequately yet. In our previous work, it was found that the positively charged surface is in favor of the demulsification process [17]. It is because the electrostatic attraction between negative oil droplets and positive surfaces accelerates the coagulation and breakage of emulsions. However, the opposite charge between pollutants and surfaces may also cause severe membrane fouling, which is not beneficial to the long-term separation process. From this point of view, intelligent membranes with reversible surface charge seem promising to obtain high separation and antifouling performance at the same time.

Other than oils, other chemicals including dyes and surfactants existing in sewage are also difficult to degrade naturally, which may cause great harm to the ecological environment and human bodies [18]. Although the membrane is widely acknowledged as a useful way for oil/water separation, it is not applicable for the interception of small molecules, leading to a high TOC value of water even after separation [19]. Thus, great effort has been devoted to contaminant removal and water purification. Currently, various approaches including physical adsorption [20,21,22], photocatalytic decomposition [23,24], oxidation [25], etc., have been employed for dye removal. For example, the membranes coated with gallic-acid-modified TiO_2_ nanoparticles are successful in separating oil-in-water emulsion and removing water-soluble contaminants. At the same time, it possesses excellent self-cleaning performance due to the photocatalytic property of TiO_2_ under UV irradiation [26]. However, the photocatalytic degradation of dyes is a time-consuming and laborious process, which largely depends on the power of the light source [27] and the nanoparticles composited to the membranes have low instability, which may become a potential source of water contamination. Additionally, most of the current studies focus on dye removal, but the capability of surfactant removal has rarely been investigated [28]. Since surfactants are unlikely to be decomposed by light, it is more difficult for us to remove them from water.

In this study, efficient oil/water separation and pollutant removal was achieved by merely controlling the surface charge of the membrane. Based on the regulation of surface charge on demulsification and antifouling performance found in our previous work, a pDMAEMA grafted PP membrane (PP-*g*-pDMAEMA) with high hydrophilicity and reversible surface charge was prepared. The interaction between the membrane and emulsions/pollutants was manipulated by the surface charges by tuning the pH. In this investigation, the emulsion separation performance of PP-*g*-pDMAEMA was studied, and the interception capability of membranes towards RB-5 and SDBS in low pH and the smart cleaning performance in high pH were discussed. To the best of our knowledge, the interception and release of both dyes and surfactants by membranes has rarely been reported. The membrane prepared in this study can be applied to oil/water separation and deep purification of water as well.

## 2. Materials and Methods

### 2.1. Materials

The PP membrane (0.22 μm) was purchased from Dalian UB Membrane Technology Co., Ltd. (Dalian, China). Diesel was purchased from a local gasoline station. 2-Isopropylthioxanthone, dopamine hydrochloride, N, N’-Methylene bis(acrylamide) (MBA) were purchased from Aladdin Company (Shanghai, China). Sodium dodecyl sulfate (SDS) and SDBS were purchased from Gaojing Company (Hanzhou, China) and Kemiou Company (Tianjin, China), respectively. RB-5 was purchased from Longsheng Company (Zhejiang, China). All the chemicals were of analytic grade and used as received without further purification.

### 2.2. The Preparation of PP-g-pDMAEMA Membrane

The preparation of the PP-*g*-pDMAEMA membrane was described in our previous work [17]. In brief, the PP membrane was first deposited with pDA to improve its hydrophilicity (PP-pDA), followed by pDMAEMA grafting under UV irradiation. The mass ratio of DMAEMA: MBA (cross-linker): ITX (initiator) was 50: 2.5: 2, and the final weight growth was approximately 8%.

### 2.3. Oil/Water Separation Experiment

The oil-in-water emulsion was prepared by adding diesel into water (v:v = 1:100) with 0.5 mg/mL SDS. The mixture was then stirred for 12 h. The obtained emulsion was stable for several days. The oil/water separation experiment was taken under the vacuumed pressure of 0.1 MPa according to our previous work [17]. The permeate was collected every 5 min, and the membrane was rinsed with Milli-Q water or alkaline buffer (pH 9.0) every 30 min. The process was repeated for three cycles and the flux was calculated with the equation:

*Flux* = *V*/(*A* × *t*), (1)

where, *F* is the flux of the membrane (L·m^−2^·h^−1^), *V* is the volume of the permeate (L), *A* is the area of the membrane (m^2^), *t* is the duration for separation (h).

### 2.4. The Adsorption and Desorption of Dyes and Surfactants

An aqueous solution of RB-5 (2.5 mg/L) and SDBS (0.5 mg/mL) was filtered by the membrane under the vacuumed pressure of 0.1 MPa. The permeate was collected in the flask for UV spectrum analysis. For the desorption process of RB-5 and SDBS, the membrane was rinsed with Milli-Q water or alkaline buffer (pH 9.0).

### 2.5. Instruments and Characterization

The surface chemistry of the membrane was characterized by an X-ray photoelectron spectrometer (Thermo Fisher Scientific, MA, USA). The wetting process of water droplets (3 μL) on the membrane surface was recorded and the contact angles were measured by a contact angle analyzer equipped with videotape (DSA20, KRUSS, Hamburg, Germany). The surface zeta potential of the membrane was investigated by a surface zeta potential analyzer (SurPASS, Anton Parr, Austria). The morphologies of the membrane were observed by a scanning electron microscope (SEM, JEOL JSM5610LV, Japan) at 5000 magnification. The UV-8000 spectrometer (Metash, Shanghai, China) was used to measure the transmittance of the permeate. The concentration of the RB-5 and SDBS solution was also determined by UV spectrometry.

## 3. Results and Discussion

**Figure 1:**
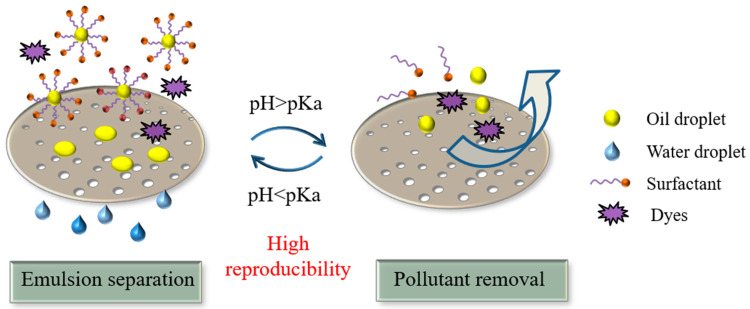
Scheme of pH-sensitive membrane for the application of emulsion separation and the removal of dyes and surfactants.

Industrial wastewater contains a large amount of small molecular pollutants with negative charges because of the extensive use of anionic surfactants and dyes. In our previous work, the influence of membrane surface charge on the oil/water emulsion separation performance and membrane fouling was systematically studied [17]. It was found that a positively charged membrane is favorable for demulsification but easy to be fouled by anionic surfactants, resulting in rapid water flux decline. In order to solve this problem, a kind of intelligent membrane with reversible surface charge responding to pH changes was prepared for emulsion separation. In this study, pDMAEMA, a pH-responsive polymer (isoelectric point is approximately pH 6.0) was grafted onto a PP membrane through photo-crosslinking technology. The obtained membrane possessed positive charges in water (weak acid environment) due to the protonation of amino groups and turned to negative when being rinsed with alkaline buffer (pH 9.0) (Figure 1). This membrane with a reversible charge is supposed to be beneficial for both demulsification and contamination removal, and therefore facilitates surface renewal during long-term application.

### 3.1. Surface Characterization of PP-g-pDMAEMA Membrane

**Figure 2:**
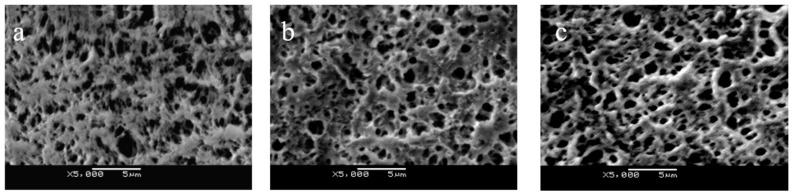
The SEM images of (**a**) PP membrane, (**b**) PP-pDA membrane, and (**c**) PP-*g*-pDMAEMA membrane.

As we know, the PP membrane has the advantages of high porosity, corrosion resistance, and high mechanical performance. However, the unmodified PP membrane is too hydrophobic for water to permeate [17]. Therefore, dopamine was used for surface modification before polymer grafting. As shown in Figure 2a, there are irregular and interconnected pores in the pristine PP membrane. The membrane presented similar surface morphologies after pDA deposition, while the PP-*g*-pDMAEMA membrane became slightly rougher with polymers on the surface (Figure 2b,c). Since the grafting rate of pDMAEMA is relatively low (~8%), the membrane still maintained a high porosity.

**Figure 3:**
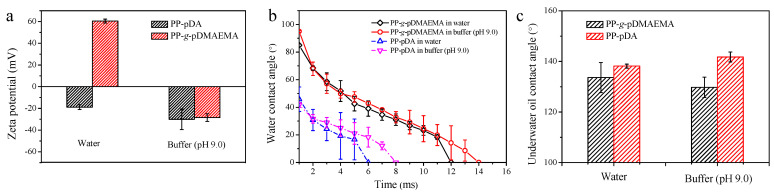
(**a**) The surface zeta potential of membranes; (**b**) the water contact angle and wicking curve of membranes; (**c**) the underwater oil contact angle of membranes in different pH 3.2. Emulsion Separation Performance of the PP-g-pDMAEMA Membrane.

To verify the switchable surface potential in different pH levels, the surface zeta potential of the membrane was measured. Due to the hydroxyl groups present in the pDA molecule, the PP-pDA membrane showed a negative charge both in water (weak acid environment) and alkaline buffer (Figure 3a). On the contrary, strong positivity (ζ≈+60 mV) was found on the surface of the PP-*g*-pDMAEMA membrane in water and the surface charge switched to negative (ζ ≈ −30 mV) in the alkaline buffer. In the latter case, the pDMAEMA chains dehydrated and collapsed due to the deprotonated amino groups. Therefore, more pDA molecules were exposed to the outermost layer, causing the surface to turn negatively charged.

The surface wettability was characterized by water contact angle (WCA) and underwater oil contact angle (UOCA) measurement. As shown in Figure 3b, the initial WCA (at t = 0 s) of the PP-*g*-pDMAEMA membrane was approximately 90°. The membrane was wetted quickly when the water droplet reached the surface, and the WCA reduced to 0° in less than 15 ms (Figure 3c). The UOCA of the membrane was larger than 130°, suggesting that the membranes were highly hydrophilic and oleophobic either in water or buffer (pH 9.0). Although the membrane wettability was unremarkably affected by pH, the PP-*g*-pDMAEMA membranes, which possessed more hydroxide groups on the surface, were more hydrophobic than the pDA deposited ones. Also, the pores of the PP-*g*-pDMAEMA membrane were partially blocked by polymers, leading to a longer wicking time [29].

**Figure 4:**
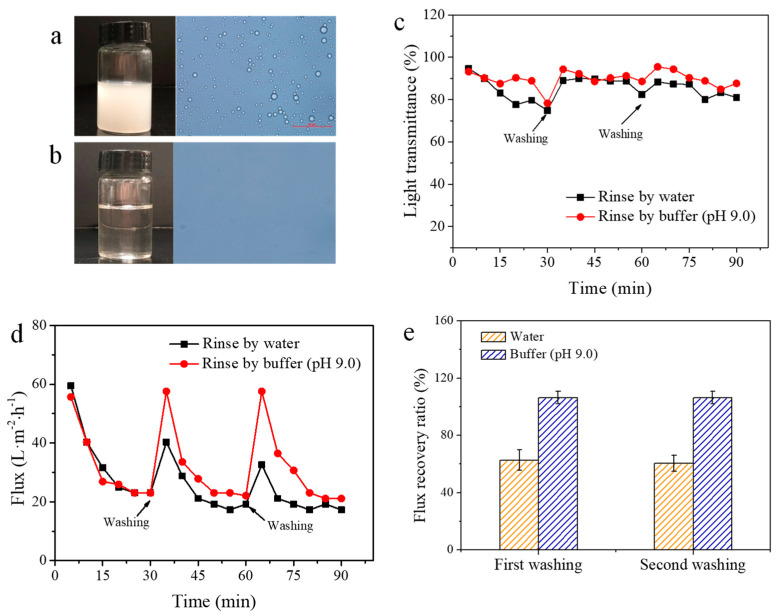
The light transmittance of emulsion (**a**) before and (**b**) after being separated by a PP-*g*-pDMAEMA membrane; (**c**) the light transmittance and (**d**) the flux of PP-*g*-pDMAEMA membranes during three cyclic separations; (**e**) FRR in the second and the third cycle.

The separation of SDS-stabilized diesel-in-water emulsions was performed to evaluate the oil/water separation performance of the membranes. The particle size of the feed emulsion, determined by a dynamic light scattering particle size analyzer (Nano-S, Malvern, England), was 400–500 nm. After being separated by the PP-*g*-pDMAEMA membrane, the mean particle size decreased to tens of nanometers. The emulsion became clear and transparent, and almost no oil droplets were observed under the optical microscope. It was suggested that most of the oil in the emulsion was removed (Figure 4a,b). After each cycle (30-min separation), the PP-*g*-pDMAEMA membrane was rinsed with water or alkaline buffer (pH 9.0). It was found that the separation efficiency was high because the light transmittance of the permeate was above 80% in both situations (Figure 4c), and the flux decreased sharply from 60 L·m^−2^·h^−1^ to about 20 L·m^−2^·h^−1^ in the first cycle (Figure 4d). For the membranes rinsed only by water, the flux reduced gradually in the second and third cycles. The flux recovery ratio (FRR) was only 60% (Figure 4e), indicating that the membranes were severely polluted by the foulants (oil droplets, SDS clusters, etc.). However, the flux recovered nearly 100% after the membrane was washed with the alkaline buffer in each cycle. It was attributed to the reduced interaction between pollutants and the negatively charged membrane in high pH. Hence, a smart cleaning performance during emulsion separation was realized in this study.

### 3.2. Adsorption and Desorption of Reactive Dyes on the PP-g-pDMAEMA Membrane

**Figure 5:**
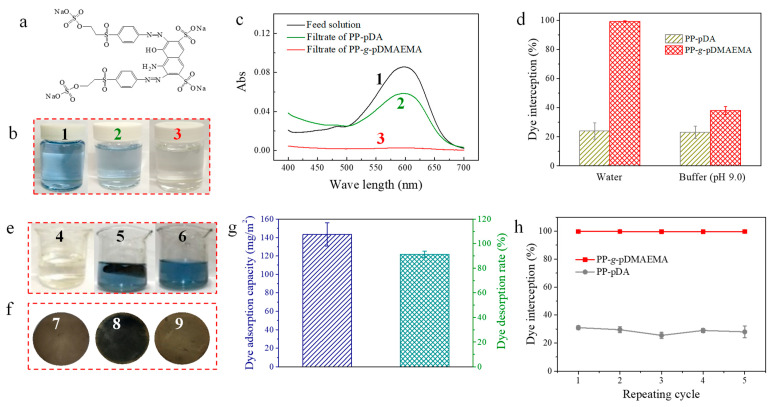
(**a**) The molecular structure of RB-5; (**b**) the photos and (**c**) UV spectrum of RB-5 feed solution (1) and filtrates (2, 3); (**d**) the dye interception rate of PP-pDA and PP-*g*-pDMAEMA membranes in different pH levels; (**e**) the photos of the solution before (4) and after (5, 6) dye desorption; (**f**) the photos of PP-*g*-pDMAEMA membranes (7), and membranes after dye adsorption (8) and desorption (9); (**g**) the dye adsorption capacity and dye desorption rate of PP-*g*-pDMAEMA membrane; (**h**) the dye interception rate of PP-pDA and PP-*g*-pDMAEMA membranes during five cyclic separations.

To evaluate the dye removal capability of the PP-*g*-pDMAEMA membrane, RB-5 was selected as a model pollutant molecule. The reactive dye can be easily hydrolyzed into anionic molecules in water (Figure 5a). Characteristic adsorption at 600 nm was found in the spectrum of the feed solution. After being separated by the PP-*g*-pDMAEMA membrane, the solution turned colorless and the characteristic peak disappeared in the spectrum (Figure 5b,c), suggesting that nearly 100% of dyes were intercepted by the membrane. The residual amount of dyes in the filtrate was analyzed by UV spectrometry. It was found that the dyes could be totally removed from the water when the initial dye concentration was lower than 12 mg/L. The adsorption capacity of the PP-*g*-pDMAEMA membrane reached 140 mg/m^2^ (Figure 5d). Dyes were intercepted by the membrane through the strong attraction between negative RB-5 molecules and positively charged pores. This property could be utilized for the deep purification of wastewater containing trace amounts of negatively charged foulants. As a comparison, the dye removal rate of PP-pDA membrane was only 25%, and that of the PP-*g*-pDMAEMA membrane in alkaline condition (pH 9.0) was as low as 40%.

Although the PP-*g*-pDMAEMA membrane is easily contaminated by dyes, most of the dyes can desorb from the membrane when being rinsed with alkaline buffer (Figure 5e,f). In pH 9.0, approximately 90% of dyes could come off from the surfaces due to the electrostatic repulsion between dyes and the membranes (Figure 5g). Furthermore, the dye adsorption and desorption processes are reversible via manipulating the ambient pH. Since the surface is renewable, the dye interception rate remained nearly 100% even after repeating for five cycles (Figure 5h). As a comparison, the RB-5 removal capability of the PP-pDA membrane stayed at a low level (approximately 30%), indicating poor dye interception performance.

### 3.3. Adsorption and Desorption of Surfactants

**Figure 6:**
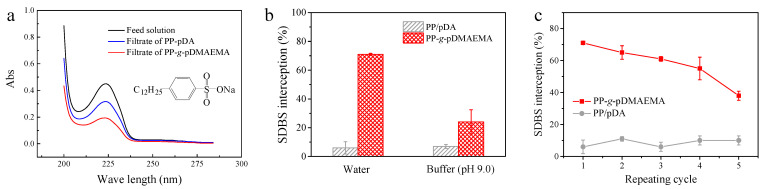
(**a**) The UV spectrum of SDBS feed solution and filtrates (the inset is the molecular structure of SDBS); (**b**) SDBS interception rate of PP-pDA and PP-*g*-pDMAEMA membranes in different pH levels; (**c**) SDBS interception rate of PP-pDA and PP-*g*-pDMAEMA membranes during five cyclic separations.

SDBS is a kind of anionic surfactant, which is frequently used in the industry. It is used as a model molecule to analyze the surfactant adsorption and desorption performance of membranes. SDBS has an adsorption of 224 nm in the UV spectrum due to the benzene ring in the molecule (Figure 6a). After being separated by the PP-*g*-pDMAEMA membrane, the UV adsorption strength of the filtrate reduced significantly. The SDBS interception rate of the PP-*g*-pDMAEMA membrane achieved approximately 70% in water, while it decreased to about 25% in pH 9.0 (Figure 6b). The efficient removal of SDBS from water is also attributed to the opposite charge between the membrane and the SDBS molecules. However, the SDBS molecule has a much smaller size than RB-5 and possesses fewer sulfate groups and benzene rings, thus the electrostatic and conjugation interaction between SDBS and the membrane is weaker. Therefore, it is more difficult for the membranes to intercept SDBS molecules. As a control, the SDBS interception rate of the PP-pDA membrane is below 10%.

After being rinsed by a buffer (pH 9.0), SDBS partially came off the PP-*g*-pDMAEMA membrane, and the SDBS interception rate recovered to some extent, indicating a relatively high smart antifouling performance. However, the value reduced from 70% to 40% after five cycles (Figure 6c). SDBS is an amphiphilic molecule with negatively charged groups in its hydrophilic end. Once they deposit onto the surface, the surface may lose its charges, causing weakened interaction between membrane and surfactants and a reduced surfactant interception capability.

## 4. Conclusions

In this study, pDMAEMA was grafted onto a PP membrane via UV-initiated polymerization and the obtained membrane presented positively in water and negative charges in an alkaline buffer (pH 9.0). Efficient separation of SDS-stabilized oil-in-water emulsions was realized by the PP-*g*-pDMAEMA membrane and the flux recovery ratio reached approximately 100% after the membrane was rinsed by an alkaline buffer (pH 9.0). The pollutant interception and resistance capability were investigated by the adsorption and desorption of dyes and surfactants. In water, the positively charged membrane assisted the demulsification of emulsions and the strong interaction between membrane and pollutant also led to significant removal of dyes and surfactants. In high pH (e.g., pH 9.0), the membrane surface charge turned to negative, resulting in the release of pollutants and the renewal of the membrane. Since reactive dyes and anionic surfactants are widely used in industry, the membrane with switchable surface charge prepared in this study shows a promising future in efficient emulsion separation with excellent fouling resistance and pollutant removal to realize the deep purification of water.

## Data Availability

Data is contained within the article.
